# The clusters of health-risk behaviours and mental wellbeing and their sociodemographic correlates: a study of 15,366 ASEAN university students

**DOI:** 10.1186/s12889-022-14233-2

**Published:** 2022-10-01

**Authors:** Apichai Wattanapisit, Hanif Abdul Rahman, Josip Car, Khadizah Haji Abdul-Mumin, Ma. Henrietta Teresa O. de la Cruz, Michael Chia, Michael Rosenberg, Moon-ho Ringo Ho, Surasak Chaiyasong, Trias Mahmudiono, Yuvadee Rodjarkpai, Ivo D. Dinov, Mohammad Ottom, Areekul Amornsriwatanakul

**Affiliations:** 1 School of Medicine, Walailak University, Nakhon Si Thammarat, Thailand; 2 Walailak University Hospital, Nakhon Si Thammarat, Thailand; 3 Institute of Health Sciences, Universiti Brunei Darussalam, Gadong, Brunei Darussalam; 4 University of Michigan, Ann Arbor, MI USA; 5 Centre for Population Health Sciences, Lee Kong Chian School of Medicine, Nanyang Technological University, Singapore, Singapore; 6 School of Nursing and Midwifery, La Trobe University, Melbourne, VIC Australia; 7 Faculty Ateneo School of Medicine and Public Health, Ateneo de Manila University, Quezon City, The Philippines; 8 Physical Education & Sports Science, National Institute of Education, Nanyang Technological University, Singapore, Singapore; 9 School of Human Sciences (Sport Science, Exercise and Health), University of Western Australia, Perth, WA Australia; 10 College of Sports Science and Technology, 999 Mahidol University, Phutthamonthon Sai 4 Rd, Salaya, Phutthamonthon District, Nakhon Pathom, 73170 Thailand; 11 School of Social Sciences, Nanyang Technological University, Singapore, Singapore; 12 Alcohol and Health Promotion Policy Research Unit, Faculty of Pharmacy, Mahasarakham University, Kantharawichai, Mahasarakham Thailand; 13 Department of Nutrition, Faculty of Public Health, Universitas Airlangga, Surabaya, Indonesia; 14 Faculty of Public Health, Burapha University, Saen Suk, Chon Buri Thailand; 15 Department of Information Systems, Yarmouk University, Irbid, Jordan

**Keywords:** Diet, Drinking, Mental wellbeing, Physical activity, Smoking, Students, University

## Abstract

**Background:**

This study investigated, through cluster analysis, the associations between behavioural characteristics, mental wellbeing, demographic characteristics, and health among university students in the Association of Southeast Asian Nations (ASEAN) University Network – Health Promotion Network (AUN-HPN) member universities.

**Methods:**

Data were retrieved from a cross-sectional self-administered online survey among undergraduate students in seven ASEAN countries. A two-step cluster analysis was employed, with cluster labels based on the predominant characteristics identified within the clusters. The ‘healthy’ cluster was assigned as the reference group for comparisons using multinomial logistic regression analysis.

**Results:**

The analytic sample size comprised 15,366 university students. Five clusters of student-types were identified: (i) ‘Healthy’ (*n* = 1957; 12.7%); (ii) ‘High sugary beverage consumption’ (*n* = 8482; 55.2%); (iii) ‘Poor mental wellbeing’ (*n* = 2009; 13.1%); (iv) ‘Smoker’ (*n* = 1364; 8.9%); and (v) ‘Alcohol drinker’ (*n* = 1554; 10.1%). Being female (OR 1.28, 95%CI 1.14, 1.45) and being physically inactive (OR 1.20, 95%CI 1.04, 1.39) increased the odds of belonging to the ‘High sugary beverage consumption’ cluster. Being female (OR 1.21, 95%CI 1.04, 1.41), non-membership in a sports club (OR 1.83, 95%CI 1.43, 2.34) were associated with ‘Poor mental wellbeing’. Obesity (OR 2.03, 95%CI 1.47, 2.80), inactively commuting to campus (OR 1.34, 95%CI 1.09, 1.66), and living in high-rise accommodation (OR 2.94, 95%CI 1.07, 8.07) were associated with membership in the ‘Smoker’ cluster. Students living in The Philippines, Singapore, Thailand, and Vietnam had a higher likelihood of being alcohol drinkers, compared with those who lived in Brunei.

**Conclusions:**

ASEAN university students exhibited health-risk behaviours that typically clustered around a specific health behaviour and mental wellbeing. The results provided support for focusing interventions on one dominant health-risk behaviour, with associated health-risk behaviours within clusters being potential mediators for consideration.

## Background

Health-risk behaviours are defined as behaviours with potentially negative effects on health such as risks of diseases and injuries [1, 2]. Health-risk behaviours vary in different age groups, environments and cultures [3–5]. Specifically, undergraduate students are in early adulthood and are in transition from high school to university. Health behaviours in university life may have a long-term impact on health conditions and incidence of chronic diseases in later adulthood [6]. Several health-risk behaviours, such as, tobacco smoking, alcohol drinking, physical inactivity, and unhealthy diet, and also mental wellbeing are highlighted as important factors of health in early adulthood [7–9]. The cited lifestyle behaviours are known risk factors for non-communicable diseases (NCDs) such as diabetes mellitus, stroke, coronary heart disease, and some forms of cancer [10]. These health-risk behaviours are potentially modifiable and preventable [11, 12]. Modification of these health-risk behaviours can improve health and reduce risks of health problems in later adulthood [13].

Apart from health care agencies, Higher Education Institutions like universities have an essential role in promoting health [14, 15]. In Southeast Asia, the Association of Southeast Asian Nations (ASEAN), consists of 10-member countries: Brunei Darussalam, Cambodia, Indonesia, Lao, Malaysia, Myanmar, The Philippines, Singapore, Thailand, and Vietnam, advocates policies that emphasise the important role that universities play in health promotion [16]. In 2014, a new thematic network of the ASEAN, the ASEAN University Network - Health Promotion Network (AUN-HPN), was established for the purpose of health promotion in the ASEAN region [17]. The AUN-HPN focuses on health promotion among university students and staff under the healthy university framework [16]. The framework includes building systems and infrastructures to support health promoting environments and covers the thematic areas as (i) zero tolerance areas (i.e., smoking, alcohol consumption, illicit drug use, gambling, violence, bullying and sexual harassment, and road safety violations) and (ii) health promotion areas (i.e., health literacy, mental wellbeing, social interaction (e.g., student clubs), physical activity and active mobility, healthy diet and balanced nutrition, safe sexual behaviour, and work-life balance and healthy ageing [15]. To achieve these targets, several strategies have been implemented, and these included health education and health promotion research [16].

Individuals may have a single dominant health-risk behaviour or multiple health-risk behaviours. Previous research classified people with health-risk behaviours into single dominant or combined health-risk behavioural clusters [7–9, 18–20]. Health-risk behaviours often co-occur or cluster, and having many health-risk behaviours concurrently could increase the probability of mortality (e.g., from cancers), and therefore these lifestyle behaviours have significant public health implications. However, information about either single or combined health behaviours, such as smoking, alcohol drinking, fruit and vegetable consumption, and sugary beverage consumption is scarce and their associations with mental wellbeing among ASEAN university students are not clear. This first surveillance of the health behaviours among ASEAN university students is thus important for understanding the situation and better-informs the health promotion strategies of the AUN-HPN. The identification of the behavioural health-risk and mental wellbeing clusters including their sociodemographic correlates, provides helpful information for designing targeted health-enabling interventions that can tackle multiple health-risk behaviours at the same time for university students. The present study foregrounded the behavioural health-risk and mental wellbeing clusters among ASEAN university students and investigated the associations between the identified clusters and student sociodemographic information.

## Methods

### Study design and data source

Data analysed were retrieved from a cross-sectional online survey, called the AUN-HPN health behavioural survey. The survey was conducted between 2020 and 2021 and investigated the health-related behaviours and mental wellbeing of ASEAN students from 17 AUN-HPN member universities across seven ASEAN countries (Table 1). The online survey comprised seven sections: 1) Physical activity, 2) Social support for physical activity, 3) University’s environment, 4) Health-related behaviours, 5) Mental wellbeing, 6) Opinion regarding university support, and 7) Sociodemographic information. The survey was developed based on previously tested instruments [21–24]. The survey, originally in English, was translated into four languages: Bahasa Indonesia, Malaysian, Thai, and Vietnamese. A pilot test of the online survey that included garnering student feedback on the survey using Qualtrics platform (Qualtrics International Inc., WA, USA) was conducted a sub-sample of university students to ensure comprehension and functionality of the online survey.

**Table 1:** Participating universities in seven ASEAN countries

Countries (n)	Total number of students n (%)	University names
Brunei Darussalam (1)	1020 (6.6)	Universiti Brunei Darussalam
Indonesia (3)	338 (2.2)	Universitas Airlangga
	3113 (20.3)	Universitas Indonesia
	979 (6.4)	Universitas Gadjah Mada
Malaysia (2)	76 (0.5)	University of Malaya
	213 (1.4)	Universiti Putra Malaysia
The Philippines (1)	322 (2.1)	Ateneo de Manila University
Singapore (1)	259 (1.7)	Nanyang Technological University
Thailand (8)	634 (4.1)	Burapha University
	253 (1.7)	Chiang Mai University
	265 (1.7)	King Mongkut’s University of Technology North Bangkok
	312 (2.0)	Naresuan University
	267 (1.7)	Mahasarakham University
	619 (4.0)	Mahidol University
	1247 (8.1)	Thammasat University
	397 (2.6)	Walailak University
Vietnam (1)	5052 (32.9)	Vietnam National University

### Measures

#### Student demographic characteristics

Demographic characteristics including year of study (year 1, 2, 3, and 4 or above); age (18 years, 19 to 21 years, and ≥ 22 years); gender (male and female); country (Brunei Darussalam, Indonesia, Malaysia, The Philippines, Singapore, Thailand, and Vietnam); body mass index (BMI) (‘underweight’ (< 18.5 kg/m^2^), ‘normal’ (18.5 to 22.9 kg/m^2^), ‘overweight’ (23.0 to 24.9 kg/m^2^), and ‘obese’ (≥25 kg/m^2^) according to World Health Organization (WHO) Asian cut-offs) [25]; the different types of grading from each institution was standardised into a 5-point grade point average (GPA) scale, which was interpreted into three levels consisting of high GPA (> 3.9), moderate GPA (3.3 to 3.9), and low GPA (≤3.2); place of living (on-campus and off-campus); commute time to campus (< 15 min, 15 to 30 min, 30 to 45 min, 45 to 60 min, 60 to 90 min, and > 90 min); commute type (active and inactive transportation); housing type (single house, townhouse, apartment, high-rise condo), and member of sports club (yes and no) were collected.

#### Exercise/sport participation, physical activity, sedentary time, and sleep

Exercise/sport participation was classified into four categories: none, 1 to 3 days/week, 4 to 6 days, and > 6 days/week.

The Global Physical Activity Questionnaire (GPAQ) version 2.0, which had an acceptable concurrent validity (*r* = 0.54) and high level of repeatability (0.67–0.81) was used to collect data on physical activity levels [26, 27]. Physical activity levels were classified into ‘inactive’ (< 600 Metabolic Equivalent (MET)-min/week) and ‘active’ (≥600 MET-min/week) [28].

Sedentary time was collected from the last item of GPAQ 2.0 and divided into three groups: < 4 hours/day, 4 to 8 hours/day, and > 8 hours/day). Sleep time were dichotomised into < 7 hours/day and ≥ 7 hours/day, based on recommendations on sleep hours per night for healthy adults (18–60 years) (i.e., 7 or more sleep hours per night) [29].

#### Smoking and alcohol drinking

Students were identified as smokers or drinkers when they reported that they are current smokers or drinkers (drink/smoke daily).

#### Fruit and vegetable consumption, snacking, sugary beverage consumption, and salt intake

Students were classified as healthy (≥5 servings/day) or unhealthy (< 5 servings/day) fruit and vegetable consumer. Students, who ate snacks or fast food every day, were categorised into at-risk snacking category; and otherwise, were categorised as at lower risk of snacking. Sugary beverage consumption was classified into at-risk consumption (drink sugary beverage every day) and lower risk of sugary beverage consumption (did not drink every day). At-risk salt intake was defined as when a student added salt to their food before eating ≥1 teaspoon/day, and at a lower risk of salt intake meant adding < 1 teaspoon/day of salt.

#### Mental wellbeing

The shortened Warwick-Edinburgh Mental Wellbeing Scale (WEMWBS) that contained seven items, is a reliable and valid tool for assessing the mental wellness of university students, was used to assess mental wellbeing [22]. The WEMWBS score was dichotomized into negative (poor) and positive (good) mental wellbeing.

### Statistical analysis

The R v4.1.1 and RStudio v1.4.1717 for Mac (RStudio, MA, USA) were used for all analyses. Incomplete survey records (i.e., missing demographic characteristics or relevant health-risk behaviours) were removed from the analysis. A two-step cluster analysis using k-means and hierarchical clustering were employed. In step-one, the number of clusters was determined using k-means algorithm, which indicated that a five-cluster model was optimal [30]. In step-two, hierarchical clustering using Euclidean distance was used to subset the data based on the five-clusters [31]. Descriptive statistics were used to present sociodemographic characteristics of the samples and characteristics of the health-risk behaviours clusters. The cluster labels were based on the predominant characteristics within the clusters. The ‘healthy’ cluster was identified based on the least number of risk factors and was used as the reference group for comparison using multinomial logistic regression analysis, which was performed to assess the associations between health-risk behaviour clusters and demographic characteristics. McFadden’s R-square was used to check for overall model fit. Statistical significance was set at *p* < 0.05.

## Results

### Sociodemographic characteristics of participants

A final sample of 15,366 ASEAN university students was used for analyses. The sample consisted about equal distribution of male (47.4%) and female (52.6%) students. A majority of the university students were from Vietnam (33.2%), followed by Indonesia (28.8%), and Thailand (25.6%). The participants were mostly in the first year (64.7%) of university life, had normal BMI (61.5%), achieved moderate GPA (69.2%), lived off-campus (65.2%), and commuted to the university using a physically inactive means of transportation (82.9%) (Table 2).

**Table 2:** Sociodemographic characteristics of the university students

Characteristics	n	%
Gender		
Male	7289	47.4
Female	8077	52.6
Age in years (*n =* 13,597)		
18	2496	18.4
19 to 21	9016	66.3
≥ 22	2085	15.3
Academic year		
1st	9940	64.7
2nd	2895	18.8
3rd	1800	11.7
4th or more	731	4.8
BMI (*n* = 13,097)		
Normal	8441	61.5
Underweight	2917	21.3
Overweight	1739	12.7
Obese	624	4.5
Country		
Brunei Darussalam	1020	6.6
Indonesia	4430	28.8
Malaysia	289	1.9
The Philippines	322	2.1
Singapore	259	1.7
Thailand	3940	25.7
Vietnam	5106	33.2
GPA (*n =* 12,151)		
≤ 3.2	2443	20.1
3.3 to 3.9	8406	69.2
> 3.9	1302	10.7
Place of living		
On-campus	5345	34.8
Off-campus	10,021	65.2
Commute time to university		
< 15 min	5917	38.5
15 to 30 min	4127	26.9
30 to 45 min	1973	12.8
45 to 60 min	1692	11.0
60 to 90 min	1059	6.9
> 90 min	598	3.9
Commute type to university		
Active	2639	17.1
Inactive	12,727	82.9
Housing type		
Single house	11,319	73.7
Townhouse	2773	18.0
Apartment	1201	7.8
High rise condo	73	0.5
Member of sports club		
Yes	9054	58.9
No	6312	41.1

### Cluster outputs and characteristics

**Fig. 1:**
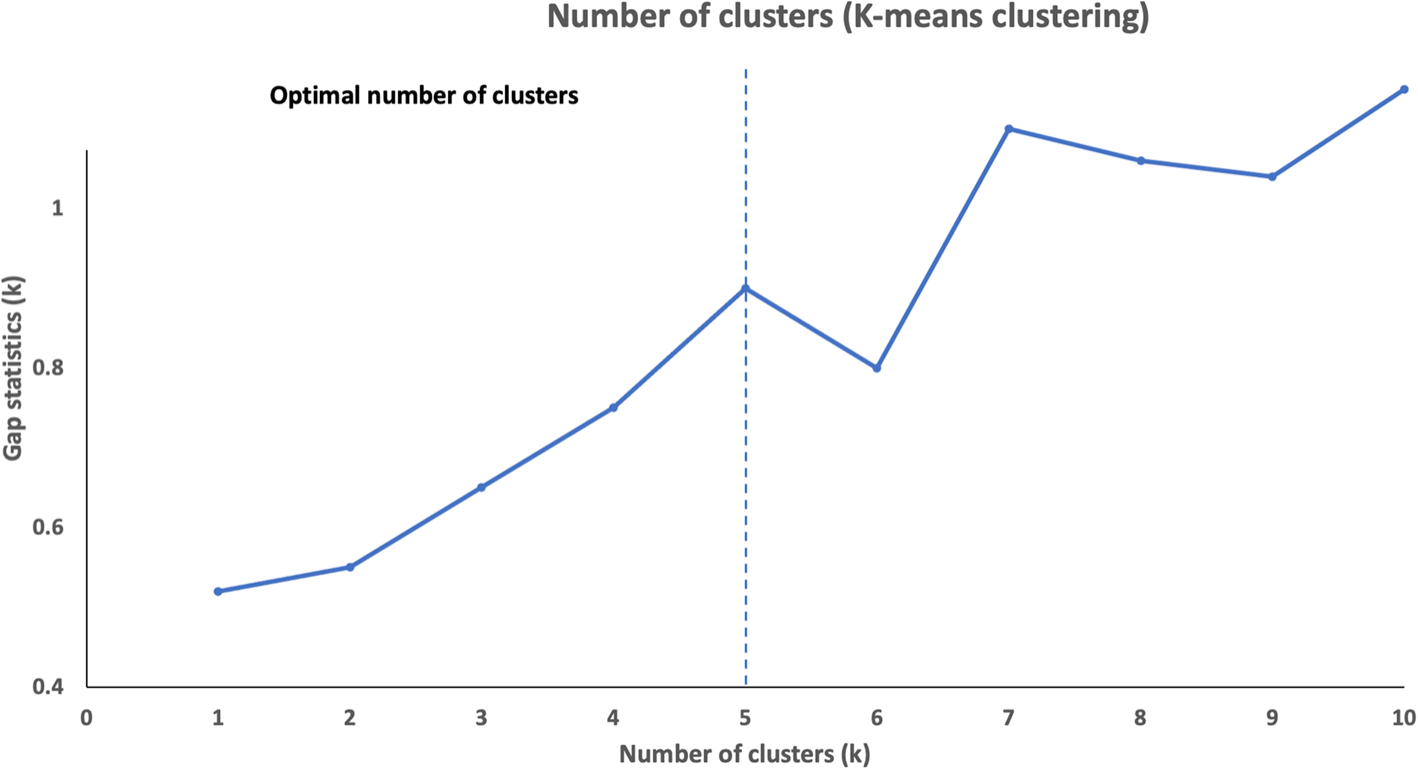
Number of clusters determined by k-means clustering method

After data cleaning, the analytical sample comprised 15,366 students. The two-step cluster analysis grouped respondents with similar health-risk behaviours and mental wellbeing resulted in 4 to 15 solutions, and it emerged that the five-cluster model was the most optimal (Fig. 1). Table 3 presents the characteristics of each cluster where the greater proportion of health-risk behaviours or mental wellbeing exhibited by the cluster determined the cluster label.

**Table 3:** Characteristics of the naturally occurring clusters extracted from the dataset (*n* = 15,366)

		Cluster 1	Cluster 2	Cluster 3	Cluster 4	Cluster 5
	Total	Healthy	High sugary beverage consumption	Poor mental wellbeing	Smoker	Alcohol drinker
	(***n*** = 15,366)	(***n*** = 1957)	(***n*** = 8482)	(***n*** = 2009)	(***n*** = 1364)	(***n*** = 1554)
Health behaviours and mental wellbeing	n (%)	n (%)	n (%)	n (%)	n (%)	n (%)
**Exercise/sport participation**						
None	2131 (13.87)	309 (15.79)	1123 (13.24)	409 (20.36)	174 (12.76)	116 (7.46)
1–3 days/week	8081 (52.59)	1111 (56.77)	4596 (54.19)	1068 (53.16)	678 (49.71)	628 (40.41)
4–6 days/week	2937 (19.11)	319 (16.30)	1548 (18.25)	260 (12.94)	330 (24.19)	480 (30.89)
> 6 days/week	2217 (14.43)	218 (11.14)	1215 (14.32)	272 (13.54)	182 (13.34)	330 (21.24)
**Physical activity**						
Inactive (< 600 MET-min/week)	6097 (39.68)	807 (41.24)	3390 (39.97)	962 (47.88)	504 (36.95)	434 (27.93)
Active (≥600 MET-min/week)	9269 (60.32)	1150 (58.76)	5092 (60.03)	1047 (52.12)	860 (63.05)	1120 (72.07)
**Sedentary time**						
< 4 hours/day	2376 (15.46)	360 (18.40)	1272 (15.00)	278 (13.84)	226 (16.57)	240 (15.44)
4–8 hours/day	6213 (40.43)	742 (37.92)	3605 (42.50)	685 (34.10)	569 (41.72)	612 (39.38)
> 8 hours/day	6777 (44.10)	855 (43.69)	3605 (42.50)	1046 (52.07)	569 (41.72)	702 (45.17)
**Sleeping hours**						
< 7 hours/day	10,566 (68.76)	1232 (62.95)	5965 (70.33)	1377 (68.54)	875 (64.15)	1117 (71.88)
≥ 7 hours/day	4800 (31.24)	725 (37.05)	2517 (29.67)	632 (31.46)	489 (35.85)	437 (28.12)
**Smoking**						
Smoker	1365 (8.88)	0	0	0	**1364 (100)**	1 (0.06)
Non-smoker	14,001 (91.12)	**1957 (100)**	**8482 (100)**	**2009 (100)**	0	**1553 (99.94)**
**Alcohol drinking**						
Drinker	2420 (15.75)	0	0	0	866 (63.49)	**1554 (100)**
Non-drinker	12,946 (84.25)	**1957 (100)**	**8482 (100)**	**2009 (100)**	498 (36.51)	0
**Fruit and vegetable consumption**						
Unhealthy (< 5 servings/day)	7339 (47.76)	908 (46.40)	4052 (47.77)	797 (39.67)	683 (50.07)	899 (57.85)
Healthy (≥5 servings/day)	8027 (52.24)	1049 (53.60)	4430 (52.23)	1212 (60.33)	681 (49.93)	655 (42.15)
**Snacking**						
At-risk (every day)	10,019 (65.20)	1098 (56.11)	5539 (65.30)	1283 (63.86)	1027 (75.29)	1072 (68.98)
Lower risk (not every day)	5347 (34.80)	859 (43.89)	2943 (34.70)	726 (36.14)	337 (24.71)	482 (31.02)
**High sugary beverage consumption**						
Yes	12,598 (81.99)	0	**8482 (100)**	1622 (80.74)	1148 (84.16)	1346 (86.62)
No	2768 (18.01)	**1957 (100)**	0	387 (19.26)	216 (15.84)	208 (13.38)
**High Salt intake**						
Yes	8305 (54.05)	804 (41.08)	4682 (55.20)	1063 (52.91)	815 (59.75)	941 (60.55)
No	7061 (45.95)	1153 (58.92)	3800 (44.80)	946 (47.09)	549 (40.25)	613 (39.45)
**Mental wellbeing**						
Negative mental wellbeing	2559 (16.65)	0	0	**2009 (100)**	290 (21.26)	260 (16.73)
Positive mental wellbeing	12,807 (83.35)	**1957 (100)**	**8482 (100)**	0	1074 (78.74)	1294 (83.27)

Bold numbers represent dominant health behaviours and mental wellbeing in each cluster

The largest cluster (*n* = 8482; 55.2%) of the sample that exhibited the highest proportion of high sugary beverage consumption was labelled as ‘High sugary beverage consumption’. The other four clusters according to size were labelled as ‘Poor mental wellbeing’ (*n* = 2009; 13.1%), ‘Healthy’ (*n* = 1957; 12.7%), ‘Alcohol drinker’ (*n* = 1554; 10.1%), and ‘Smoker’ (*n* = 1364; 8.9%). The ‘Healthy’ cluster exhibited mostly safe health behaviours and was used as the reference group for multinomial logistic regression. The healthy, high sugary beverage consumption, poor mental wellbeing and alcohol drinker clusters were each comprised entirely of non-smokers, with those who smoked clustering into their own smoking cluster. The healthy, high sugary beverage consumption and poor mental wellbeing clusters comprised all non-alcoholic drinkers, with the alcohol drinkers comprising all drinkers and 63% of the smoking group cluster also drinking alcohol. Members of the healthy cluster did not drink sugary beverages, and all had positive mental wellbeing. Members of the high sugary beverage consumption cluster were also non-smokers, non-drinkers and had positive mental wellbeing. Members of the smoking cluster also had high sugary beverage consumption with almost 80% having positive mental wellbeing. Members of the alcohol cluster were all non-smokers with 87% with high sugary beverage consumption, 83% positive mental wellbeing and 72% in the high physically active group. The level of exercise/sport participation, physical activity, sedentary time, sleeping hours and high salt intake did not appear to differentiate clusters.

### Associations between clusters and demographic characteristics

To further understand the similarities and differences of the 5 clusters, multinomial logistic regression analyses were run with the demographic characteristics as the predictors and the cluster membership as the outcome variable. The ‘Healthy’ cluster was chosen as the reference group in the analysis. Table 4 shows the likelihood of university students falling into the unhealthy or health-risk clusters.

**Table 4:** Factors associated with the healthy cluster (Cluster 1) using multinomial logistic regression analysis (*n* = 15,366)

Demographic characteristics	Cluster 2 High sugary beverage consumption OR (95%CI)	Cluster 3 Poor mental wellbeing OR (95%CI)	Cluster 4 Smoker OR (95%CI)	Cluster 5 Alcohol drinker OR (95%CI)
**Gender (Ref. = Male)**				
Female	1.28 (1.14, 1.45)**	1.21 (1.04, 1.41)*	0.93 (0.78, 1.10)	1.14 (0.96, 1.36)
**Age (years) (Ref. = 18)**				
19 to 21	0.94 (0.81, 1.10)	0.99 (0.81, 1.22)	1.06 (0.84, 1.34)	1.29 (0.98, 1.70)
≥ 22	0.90 (0.70, 1.15)	0.76 (0.55, 1.05)	1.09 (0.77, 1.54)	1.24 (0.85, 1.81)
**Year of study (Ref. = Year 1)**				
Year 2	1.24 (1.04, 1.46)*	1.24 (1.01, 1.52)*	1.37 (1.09, 1.72)**	1.28 (1.03, 1.60)*
Year 3	1.28 (1.01, 1.63)*	1.15 (0.85, 1.55)	1.19 (0.86, 1.64)	1.46 (1.08, 1.98)*
Year 4 or above	1.36 (0.92, 2.03)	1.37 (0.84, 2.25)	1.22 (0.72, 2.06)	1.57 (0.96, 2.59)
**BMI (Ref. = Normal)**				
Underweight	0.85 (0.74, 0.99)*	0.74 (0.61, 0.90)**	1.09 (0.86, 1.37)	1.01 (0.81, 1.25)
Overweight	0.82 (0.68, 0.97)*	0.87 (0.70, 1.09)	1.58 (1.23, 2.04)**	1.22 (0.95, 1.56)
Obese	0.91 (0.72, 1.17)	1.01 (0.75, 1.36)	2.03 (1.47, 2.80)**	0.99 (0.70, 1.40)
**Country (Ref. = Brunei Darussalam)**				
Indonesia	0.86 (0.63, 1.16)	0.34 (0.24, 0.49)**	1.17 (0.78, 1.75)	0.75 (0.40, 1.42)
Malaysia	0.45 (0.24, 0.77)**	0.32 (0.16, 0.67)**	0.23 (0.09, 0.57)**	0.20 (0.04, 0.98)*
Philippines	0.38 (0.24, 0.69)**	0.40 (0.24, 0.67)**	1.05 (0.58, 1.89)	3.11 (1.47, 6.56)**
Singapore	1.11 (0.58, 2.12)	0.91 (0.43, 1.92)	1.23 (0.53, 2.88)	14.70 (6.28, 34.42)**
Thailand	1.71 (1.16, 2.52)**	0.95 (0.60, 1.52)	1.18 (0.70, 1.99)	7.06 (3.64, 13.70)**
Vietnam	1.32 (0.98, 1.77)	0.62 (0.44, 0.86)**	0.86 (0.58, 1.30)	6.34 (3.51, 11.43)**
**GPA (Ref. = ≤ 3.2)**				
3.3 to 3.9	1.05 (0.90, 1.23)	0.97 (0.80, 1.18)	0.85 (0.69, 1.05)	0.76 (0.62, 0.92)**
> 3.9	0.91 (0.72, 1.14)	0.91 (0.68, 1.22)	0.72 (0.52, 1.01)	0.64 (0.48, 0.86)**
**Place of living (Ref. = On-campus)**				
Off-campus	1.18 (0.94, 1.49)	1.05 (0.79, 1.40)	1.10 (0.78, 1.56)	0.91 (0.67, 1.24)
**Commute time (Ref. = less than 15 min)**				
15 to 30 min	1.06 (0.92, 1.24)	1.04 (0.86, 1.26)	1.06 (0.86, 1.31)	1.04 (0.85, 1.27)
30 to 45 min	1.07 (0.88, 1.30)	1.33 (1.05, 1.70)*	1.40 (1.08, 1.82)*	1.04 (0.78, 1.38)
45 to 60 min	0.84 (0.69, 1.01)	0.83 (0.65, 1.07)	0.81 (0.61, 1.08)	0.76 (0.57, 1.02)
60 to 90 min	0.96 (0.76, 1.20)	0.95 (0.71, 1.27)	0.76 (0.53, 1.07)	0.57 (0.39, 0.83)**
> 90 min	0.88 (0.67, 1.16)	1.27 (0.90, 1.79)	0.71 (0.46, 1.10)	0.55 (0.32, 0.94)*
**Commute type (Ref. = Active)**				
Inactive	1.20 (1.04, 1.39)*	1.13 (0.93, 1.36)	1.34 (1.09, 1.66)**	1.05 (0.85, 1.30)
**Housing type (Ref. = Single house)**				
Townhouse	0.82 (0.68, 1.00)*	0.80 (0.63, 1.02)	1.03 (0.80, 1.33)	1.12 (0.89, 1.40)
Apartment	1.01 (0.81, 1.26)	0.92 (0.70, 1.22)	1.28 (0.95, 1.73)	1.25 (0.93, 1.68)
High rise condo	1.18 (0.48, 2.88)	1.20 (0.42, 3.44)	2.94 (1.07, 8.07)*	2.34 (0.78, 6.97)
**Member of sports club (Ref. = Yes)**				
No	0.99 (0.83, 1.18)	1.83 (1.43, 2.34)**	0.45 (0.35, 0.56)**	0.70 (0.54, 0.90)**

*95%CI* 95% Confidence interval, *BMI* Body Mass Index, *GPA* Grade Point Average, *OR* Adjusted odds ratio*, Ref.* Reference group

*p < 0.05, **p < 0.01

Students in Year 2 (OR 1.24, 95%CI 1.04, 1.46) and Year 3 (OR 1.28, 95%CI 1.01, 1.63), being female (OR 1.28, 95%CI 1.14, 1.45) living in Thailand (OR 1.71, 95%CI 1.16, 2.52), and being inactive (OR 1.20, 95%CI 1.04, 1.39) had a higher likelihood of falling into ‘High sugary beverage consumption’ cluster (cluster 2), compared to the ‘Healthy’ cluster. Meanwhile, students living in The Philippines (OR 0.38, 95%CI 0.24, 0.69), being underweight (OR 0.85, 95%CI 0.74, 0.99) or overweight (OR 0.82, 95%CI 0.68, 0.97), and staying in a townhouse (OR 0.82, 95%CI 0.68, 1.00) had less likelihood of being in the ‘High sugary beverage consumption’ cluster.

In comparison to the healthy group, being in 2nd year (OR 1.24, 95%CI 1.01, 1.52), females (OR 1.21, 95%CI 1.04, 1.41), non-membership to a sports club (OR 1.83, 95%CI 1.43, 2.34), and travelling between 30 to 45 minutes to campus (OR 1.33, 95%CI 1.95, 1.70) were significantly more likely to fall into the ‘Poor mental wellbeing’ cluster (cluster 3). Students who were underweight (OR 0.74, 95%CI 0.61, 0.90) and resided in Indonesia (OR 0.34, 95%CI 0.24, 0.49), Malaysia (OR 0.32, 95%CI 0.16, 0.67), The Philippines (OR 0.4, 95%CI 0.24, 0.67), or Vietnam (OR 0.62, 95%CI 0.44, 0.86) compared to students from Brunei were less likely to be in the ‘Poor mental wellbeing’ cluster.

Belonging to the ‘Smoker’ cluster (cluster 4), was associated with being in 2nd year (OR 1.37, 95%CI 1.09, 1.72), overweight (OR 1.58, 95%CI 1.23, 2.04), obese (OR 2.03, 95%CI 1.47, 2.80), travelling 30–45 minutes to campus (OR 1.40, 95%CI 1.08, 182), inactively commuting to campus (OR 1.34, 95%CI 1.09, 1.66), and living in high-rise accommodation (OR 2.94, 95%CI 1.07, 8.07) compared to members of the healthy cluster.

The alcohol drinker cluster (cluster 5) was associated with being in 2nd year (OR 1.28, 95%CI 1.03, 1.60) or 3rd year (OR 1.46, 95%CI 1.08, 1.98) compared to the healthy cluster and 1st year students. Students living in The Philippines (OR 3.11, 95%CI 1.47, 6.56), Singapore (OR 14.70, 95%CI 6.28, 34.42), Thailand (OR 7.06, 95%CI 3.64, 13.70), or Vietnam (OR 6.34, 95%CI 3.51, 11.43) had higher odds of being a drinker compared with students living in Brunei. Students with a higher GPA were less likely to belong to the alcohol cluster (OR 0.76, 95%CI 0.62, 0.92 for GPA 3.3 to 3.9; and OR 0.64, 95%CI 0.48, 0.86 for GPA > 3.9) compared to students in the healthy cluster. Students who spent more time travelling to campus (> 60 min) were less likely to be in the alcohol drinker cluster (OR 0.57, 95%CI 0.39, 0.83 for 60 to 90 min; and OR 0.55, 95%CI 0.32, 0.94 for > 90 min) compared to students in the healthy cluster. Students who were not members of a sports club had 30% lower odds of belonging to the alcohol drinker cluster than the ‘healthy’ cluster and students who belonged to a sports club.

## Discussion

The research investigated the health-risk behaviours and mental wellbeing among university students in seven ASEAN countries and it emerged that five behavioural clusters (i.e., healthy, high sugary beverage consumption, smoker, and alcohol drinker) and mental wellbeing, were identified. The results showed that among ASEAN university students, consuming sugary beverages was the dominant health behaviour across all clusters. This finding is consistent with the findings reported by Pengpid and Pletzer (2019) who observed that a high proportion of ASEAN university students consumed sugary soft drinks and this was associated with other unhealthy behaviours, such as smoking and drinking alcohol [32]. The present finding added to a small body of evidence linking the consumption of sugary beverages with a range of unhealthy behaviours among ASEAN university students. Therefore, it may be important to consider sugary beverage consumption behaviour when targeting other health-risk behaviours for improvement.

This research showed that several characteristics of university students were related to health-risk behaviours and mental wellbeing. Students in the second year of university study had a higher likelihood of having poor mental wellbeing and falling into the ‘high sugary beverage consumption’, ‘smoker’, and ‘alcohol drinker’ clusters, compared to the ‘healthy’ cluster. These results were in line with the findings of a Chinese study that showed that second year students suffered relatively higher levels of depression and stress, compared to students in the first, third, and fourth years of university study [33]. Another study conducted in Korea also supported the present results, where Korean university students in the second year experienced more mental health problems than the students in other years of study [34]. The academic demands of year 2 of university life might present greater stress and mental health challenges for year 2 students. Many universities usually set general courses for freshmen and introduce more specialised courses from the second year onwards [33]. Higher levels of stress, coupled with mental health challenges in year 2 students may relatively predispose them to getting involved in other health-risk behaviours compared to year 1 students.

Our results showed that more than half of ASEAN university students consumed sugary beverages. Paradoxically, both underweight and overweight students were less likely to consume sugary beverages compared to university peers of normal body weight. Our findings contrasted with those of a previous study on Saudi Arabian adolescents showing that intake of sugar-sweetened carbonated beverage was positively associated with BMI [35]. It appears that both socio-economic and environmental factors, are associated with high sugar diets and sugary beverage consumption in university students [36–38]. Of interest, university students’ perceptions regarding sugar intake were explored in a qualitative study and although students perceived that excessive sugar intake affected their body image because of weight-gain, the students, nonetheless thought that they were not at risk of negative health outcomes as they were young [39]. Our findings alluded that the strategies to reduce sugary beverage consumption so as to reduce obesity among ASEAN university students should be applied to all students regardless of their body weight status.

The present study revealed that poor mental wellbeing was the second most prevalent health-risk characteristic among university students. Mental health issues among university students increased the burden on campus counselling resources, and have received great attention from educators within the AUN-HPN as poor mental wellbeing could lead to significant psychological problems and tragedies (i.e. suicide) [40]. We found that students who were not a member of any sports clubs had 83% increased risk of poor mental wellbeing. Non-membership to a sports club presented the highest odds of having poor mental health, albeit be it this is only an association and may not causative. Nonetheless, mental health promotion and helping students become physically active on a regular basis are recommendations for improvement in mental wellbeing among students [40–42]. A US-based study showed that sports club participation in college students is associated with positive health-related outcomes [41]. Also aligned, Australian students with higher sports club involvement have a positive and significant association with social-emotional wellbeing indicators, such as happiness, resilience, and body image, whereas low involvement in sports club is associated with a greater incidence of mental health diagnosis [43]. There is compelling evidence for the benefits of sports involvement and participation in university students. Involvement in team sports is also associated with reduced depressive mood because sport participation protects students against social isolation [44, 45]. Other research shows that lower depression scores are reported in the moderate sports involvement group, compared to the low sports involvement group [46]. Students who are active in sports clubs have a better self-concept because sports participation helps students build confidence, acquire competent behaviours such as social skills, and release energy and aggression in socially accepted ways [47]. Joining and playing on team sports is also associated with greater life satisfaction, higher self-image, and less distress than students who are not involved in sport [48–50]. By being a member of a sports club, students are more psychologically resilient, confident, assertive, have better social skills, self-esteem, self-efficacy, self-control, self-concept, and competent [49]. Participating in sports clubs can be a means of improving mental wellbeing. Therefore, encouraging students to join sports clubs might be considered as part of strategies to promote mental wellbeing among the ASEAN university students.

Participation in sports clubs also seemed to influence the likelihood of being smokers or drinkers among ASEAN university students. The present study showed that not being sports club members was associated with less likelihood to smoke and drink alcohol. These results are consistent with evidence where university students who joined sports groups or organisational sports are more likely to smoke and drink alcohol [51–54]. A systematic review by Lisha et al. showed that university students in sports clubs reported higher levels of drinking and smoking than those who were not in any sports clubs [55]. Plausible explanations for these observations are that university students were in a transition period of their lives and were exposed to substantial changes in terms of environmental (living on their own away from families, gaining independence) and social aspects (making new friends with a need to belong). This kind of adolescent-adulthood transition is often associated with an increase in heavy and risky alcohol use [56, 57]. Theory and empirical findings suggest that peer pressure is a combination of three distinct influences: overt offers of alcohol, modelling, and social norms [58]. Overt offers of alcohol can range from polite gestures to intense goading or commands to drink [58]. Modelling occurs when the student’s behaviour corresponds to another student’s concurrent drinking behaviour [58]. Perceived social norms can serve to make excessive alcohol use appear common and acceptable to the students [58]. In the present case, making a recommendation for university students to join a sports club may seem contradictory since on the one hand, it could safeguard mental wellbeing, yet on the other hand, this could expose students to pressures and risks of high drinking and smoking. Therefore, comprehensive strategies that address these paradoxical observations and promote health among ASEAN university students should be considered. For instance, having trained student ambassadors in sports clubs and incentivising healthier activities among sports teams that are in keeping with the culture and context of each country are ideas that are worthy of exploration.

In the present research, the country of residence was associated with specific health-risk behaviours such as alcohol drinking. University students in all countries except Indonesia and Malaysia had higher odds of being current drinkers, whereas university students in Indonesia and Malaysia had lower odds, compared to those in Brunei. A majority of the population in Brunei, Malaysia, and Indonesia is Islamic and alcohol drinking is considered haram (prohibited or sinful) for consumption and therefore alcohol consumption is not likely to be high [59]. Research shows that alcohol consumption is associated with religion [60]. Moreover, some research suggested that religious commitment among the pious and those who are faithful to the teachings of religion (e.g. advocating abstinence from alcohol consumption), is associated with reduced likelihood of alcohol drinking [61]. We are of the view that differences in culture and context within each country in ASEAN pose challenges in the implementation of a common intervention programme to address alcohol consumption in university students. Further research is required to tease out factors that might play a role in alcohol consumption among university students in each country and how these county and culture-specific contexts might be built into future interventions to reduce alcohol abuse for positive results to be accomplished and sustained.

Overall, our results showed that, during the COVID-19 pandemic, only 12.7% of university students exhibited multiple healthy behaviours, with over 85% exhibiting at least one dominant unhealthy behaviour (e.g., high sugary beverage consumption, alcohol consumption and smoking). These results showed that a majority of ASEAN university students was at risk of developing multiple health problems, if their present health behaviours are not improved over time. The AUN-HPN Healthy University Framework highlighted smoking, alcohol consumption, and mental wellbeing as important areas to address [16]. A majority of smokers also drank alcohol. Health promotion policies and interventions targeting smoking behaviours should combine strategies to prevent or reduce alcohol drinking concomitantly. In need of urgent attention and action is to reduce the consumption of sugary beverages, as this habit is most prevalent among other identified health-risk behaviours and should be considered as a priority health promotion issue among ASEAN university students. Although cause-and-effect cannot be determined in the associations between the cluster of high sugary consumption and other health-risk behaviour clusters identified in our cross-sectional analyses, the results suggested that clusters of health-risk behaviours tend to ‘reinforce’ each other, and future research should examine if moderating high sugary consumption among university students might have a significant impact on other health-risk behaviours.

This cross-sectional descriptive research provided some insights into the potential areas of concern and actions for health promotion among university students in the seven ASEAN countries. First, university policy makers should focus on the transition from the 1st to 2nd year of university study. This period provides an opportunity for health advocates to intervene and prevent adolescents transiting into early adulthood from adopting high risk and unhealthy behaviours and habits. Data from the present research provided useful finer-grain information and specificity in terms of areas for intervention. Specifically, health promotion interventions should be targeted at reducing sugary beverage consumption and relatively poor mental health, especially among female students. To prevent and reduce the prevalence of smoking, interventions should focus on students who are overweight and obese as well as those who travelled to the campus by motorised transport. Some consideration and attention are needed to attend to alcohol drinking in students living in the non-Islamic countries to reduce the likelihood of alcohol abuse. While mental health may be safeguarded by joining sports clubs, the risks of picking up unhealthy behaviours of alcohol consumption and smoking in sports clubs also need to be moderated and monitored. The key results of our study established some demographic characteristics with specific health-risk behaviour and showed that health-risk behaviours were clustered and co-existed with each other. Policy makers should formulate health-enabling programmes that can best address multiple health risk behaviours at the same time such as adopting a socio-ecological model interventionist approach [62].

The present research had a number of strengths. Firstly, the research was conducted in seven countries in the Southeast Asian region and involved a sizeable sample. Secondly, the use of a self-reported online questionnaire allowed more university students to be polled in the COVID-19 pandemic when online interactions and travel restrictions were the norm. The research also had two notable limitations. The research design was cross-sectional and descriptive in nature and therefore only associations among demographic characteristics and health-risk behaviours could be established and cause-and-effect among the associations cannot be determined. Causal inference may require future research based on the longitudinal monitoring of university students. Another potential limitation was that the research was conducted during the COVID-19 pandemic, which could have a disproportionate impact on many health behaviours.

## Conclusion

The majority of ASEAN university students exhibited one common health-risk behaviour. A large proportion of the university students had a habit of sugary beverage consumption. This was followed by poor mental coping strategies, alcohol drinking, and tobacco smoking. The transition from the 1st to 2nd year of university life was particularly challenging and 2nd year university students had higher odds of exhibiting several health-risk behaviours. Health promotion strategies for healthy universities should focus on comprehensive interventions in addressing the dominant health-risk behaviour as well as the other associated health-risk behaviours within the identified clusters.

## Data Availability

The datasets generated and/or analysed during the current study are not publicly available due to restrictions on intellectual property regulations of the funding organization. Data are however available provided that an application is submitted at info@thaihealth.or.th or areekulk@gmail.com and approved by the dataset custodians. No administrative permissions were required to access raw data.

## Abbreviations

ASEAN: Association of Southeast Asian Nations
AUN-HPN: ASEAN University Network - Health Promotion Network
BMI: Body mass index
COVID-19: Coronavirus disease 2019
GPAQ: Global Physical Activity Questionnaire
GPA: Grade point average
NCDs: Non-communicable diseases
WEMWBS: Warwick-Edinburgh Mental Wellbeing Scale
WHO: World Health Organization
